# UniPET: a unified approach for whole-body CT to PET translation

**DOI:** 10.3389/frai.2026.1919564

**Published:** 2026-10-01

**Authors:** Francesco Di Feola, Valerio Guarrasi, Mikael Johansson, Paolo Soda

**Affiliations:** 1 Department of Diagnostics and Intervention, Radiation Physics, Biomedical Engineering, Umeå University, Umeå, Sweden; 2 Unit of Artificial Intelligence and Computer Systems, Department of Engineering, Università Campus Bio-Medico di Roma, Rome, Italy; 3 Department of Diagnostics and Intervention, Oncology, Umeå University, Umeå, Sweden

**Keywords:** curriculum learning, generative adversarial networks, image-to-image translation, medical image synthesis, medical imaging

## Abstract

Positron emission tomography (PET) provides critical metabolic information for oncological imaging, yet its use is constrained by radiation exposure, cost, and limited availability. Synthesizing PET-like images from computed tomography (CT) has been proposed as a way to approximate metabolic information; however, existing approaches either fail to capture region-specific variability or rely on multiple organ-specific models that do not scale to whole-body imaging. In this work, we introduce UniPET, a unified approach for whole-body CT-to-PET translation based on curriculum learning. By progressively incorporating anatomical regions of increasing complexity, UniPET enables a single network to learn coherent morpho-metabolic relationships across different regions. We evaluate UniPET on a public dataset of 900 patients and an external cohort of 579 patients. The model achieves image quality and metabolic consistency comparable to region-specific approaches, while improving over conventional whole-body models and maintaining stable performance under distribution shifts. By capturing region-specific variability within a single model, UniPET provides a promising approach for augmenting CT-based workflows with synthetic metabolic information, with potential applications in screening triage, in low-resource environments where PET is unavailable, and as an additional input for multimodal analysis pipelines. The code is available at: https://github.com/arco-group/UniPET.

## 1 Introduction

Computed Tomography (CT) and Fluorodeoxyglucose Positron Emission Tomography (FDG-PET, shortly PET in the following) play a central role in oncological diagnosis, providing distinct yet complementary information on anatomical structure and metabolic activity, respectively. Indeed, CT alone has shown limitations in accurately characterizing high-risk indeterminate pulmonary nodules detected during screening and in evaluating nodal and distant metastases for cancer staging (Fletcher et al., 2008; Groheux et al., 2016). In contrast, FDG-PET has been shown to improve diagnostic accuracy, particularly in differentiating benign from malignant lung nodules (Garcia-Velloso et al., 2016; Shim et al., 2005), and has proven highly sensitive for the evaluation of indeterminate nodules identified at screening (Veronesi et al., 2015).

However, PET use is constrained by its higher radiation exposure, which raises concerns for vulnerable populations such as pregnant women, children, and oncology patients requiring repeated imaging (Bosch de Basea et al., 2023). Moreover, such scanners are expensive, complex to maintain, and their limited availability contributes to substantial global disparities in access to functional imaging. These limitations have driven interest in alternative solutions that can provide functional imaging without the associated radiation burden and cost. A possible approach consists of generating PET radiotracer uptake from CT x-ray attenuation data using imaging synthesis using deep learning, learning the correspondence between anatomical structures in CT and metabolic activity captured in PET. In this context, image-to-image translation provides a natural framework, as it converts an image from a source domain *X* to a target domain *Y* (Pang et al., 2021) by learning the underlying cross-domain mapping and uncovering latent structural correspondences. To support the chance to learn such a CT-to-PET mapping, some studies have reported a certain degree of correlation between CT attenuation values and PET tracer uptake, either showing direct associations between tissue density and radiotracer accumulation (Giesel et al., 2017), or revealing feature-level dependencies between structural and metabolic descriptors (Bianconi et al., 2019; Esfahani et al., 2022), suggesting that learning-based approaches could exploit such high-order dependencies to bridge the gap between the two modalities. In this setting, synthetic PET should not be interpreted as a replacement for acquired PET, but rather as a probabilistic estimate of plausible metabolic patterns inferred from CT data. As such, it may provide complementary functional cues that are useful in specific scenarios, for example to support patient triage by highlighting regions that may warrant further investigation, or as an additional input for downstream algorithms, such as detection, segmentation, or risk stratification models. More broadly, it may enhance CT-based workflows in environments where PET is unavailable or impractical, or act as a preliminary step to guide the need for further imaging. To the best of our knowledge, in the literature, there exist three works that have pioneered CT-to-PET image translation (Salehjahromi et al., 2024; Nguyen et al., 2025; Guarrasi et al., 2025). In Salehjahromi et al. (2024), the authors introduced a conditional GAN framework to translate 2D CT slices into the corresponding PET slices. The method was designed in a supervised setting using paired data and focused on lung cancer thoracic imaging. Similarly, in Nguyen et al. (2025), the authors propose a conditional diffusion model that learns the mapping from bidimensional lung CT slices to the corresponding PET slices in a shared latent space.

Such two works overlook the inherently three-dimensional nature of medical imaging and does not account for the whole-body acquisitions typical of clinical PET studies, which are routinely performed to assess metastatic spread. Furthermore, whole-body CT-to-PET translation poses additional challenges due to the large image volumes and substantial anatomical variability across body regions. To address this challenge, in Guarrasi et al. (2025) we recently introduced a region-wise approach that learns the image translation mapping using one generative model per anatomical region, which are the head, trunk, arms, and legs. Their output is then combined to synthesize a whole-body PET image. Furthermore, to learn the 3D nature of the data, while coping with the related computational complexity, we adopted a patch-based training strategy: beyond reducing memory requirements, this choice mitigates data scarcity by increasing the number of training samples and enabling more robust learning from limited datasets. We experimentally observed that such a method outperforms the image synthesis of a single generative network trained using whole-body scans, albeit at the cost of increased computational demand and architectural complexity. However, extending this region-based approach to finer anatomical granularity would require a proportional increase in the number of translation networks, thus making the approach computationally impractical at scale.

Inspired by the progressive learning process observed in human training, such as radiologists who gradually move from anatomically simpler and more homogeneous cases to structurally complex and heterogeneous ones, we hereby introduce UniPET, a generative approach for 3D, whole body CT-to-PET translation that progressively learns from simpler to more complex mappings across different anatomical regions. Indeed, we design the UniPET training strategy to organize anatomical regions according to their complexity, defined in terms of spatial extent and structural variability, both of which influence the heterogeneity of the CT-to-PET mapping. To this end, we leverage curriculum learning (Bengio et al., 2009) to structure the training process, enabling the network to gradually acquire region-specific representations while preserving global spatial coherence between the anatomical input and the synthesized metabolic output. Our contributions can be summarized as follows:

- We present UniPET, a novel whole-body CT-to-PET translation framework that employs a single network trained across multiple anatomical regions.
- We design a two-stage training strategy, consisting of coarse translation and refined translation. In the first stage, the network is pre-trained on the global whole-body CT-to-PET mapping; in the second stage, curriculum learning is applied with a scheduling policy that progressively introduces anatomical regions according to their complexity, defined by a difficulty score.
- We conduct extensive experiments on a large public dataset comprising 900 patients and on an independent external cohort of 579 patients, demonstrating that UniPET achieves superior performance compared with state-of-the-art methods, in both image-level similarity metrics and clinically relevant PET-derived quantitative biomarkers on manually annotated lesions.

## 2 Methods

Let *x* denote a whole-body CT scan and *y* its corresponding PET scan so that (*x, y*)∈Γ where Γ={(x,y)|x∈X,y∈Y} is a paired dataset including CT scans and their aligned PET counterparts. The task of CT-to-PET translation is formulated as learning a mapping function *f* such that:

$$\begin{array}{l} ŷ=f\left(x\right)\approx y \end{array}$$

where ŷ denotes the synthetic PET volume generated from the CT input *x*. Our UniPET approach is shown in Figure 1, and it consists of three sequential stages designed to progressively structure the learning process. In the first stage (Figure 1a), we employ a pretrained CT segmentator *S* (Sundar et al., 2022) to extract anatomical masks corresponding to 14 distinct body regions: legs, arms, liver, lungs, brain, stomach, pancreas, spleen, kidneys, trachea, bladder, thyroid, gallbladder, and adrenal glands. In the second stage (Figure 1b), the segmented regions are grouped into subsets Γ_*k*_ using hierarchical clustering, based on two region-level features that characterize the degree of local intensity fluctuation and serve as proxies for texture complexity. These features are: (i) the intensity dynamic range, defined as the difference between the maximum and minimum voxel values within each region, and (ii) the intensity variance, which quantifies local intensity variability. The latter provides a simple yet effective measure of texture that reflects tissue heterogeneity, which in turn is closely associated with the biological characteristics of the tumor and is therefore crucial for diagnosis and prognosis in oncology (Di Feola et al., 2025). Assuming that larger anatomical regions are more challenging to model due to their greater spatial extent and structural variability, the resulting clusters are ranked by their volume. In the third stage (Figure 1c), a GAN-based CT-to-PET translation model is trained using a curriculum learning strategy. A data scheduler progressively introduces anatomical subsets into the training process according to their increasing complexity, allowing the model to first learn robust mappings in simpler regions and subsequently generalize to more complex ones. This gradual learning scheme enhances both convergence stability and translation quality. The individual components of UniPET are described in detail below.

**Figure 1:**
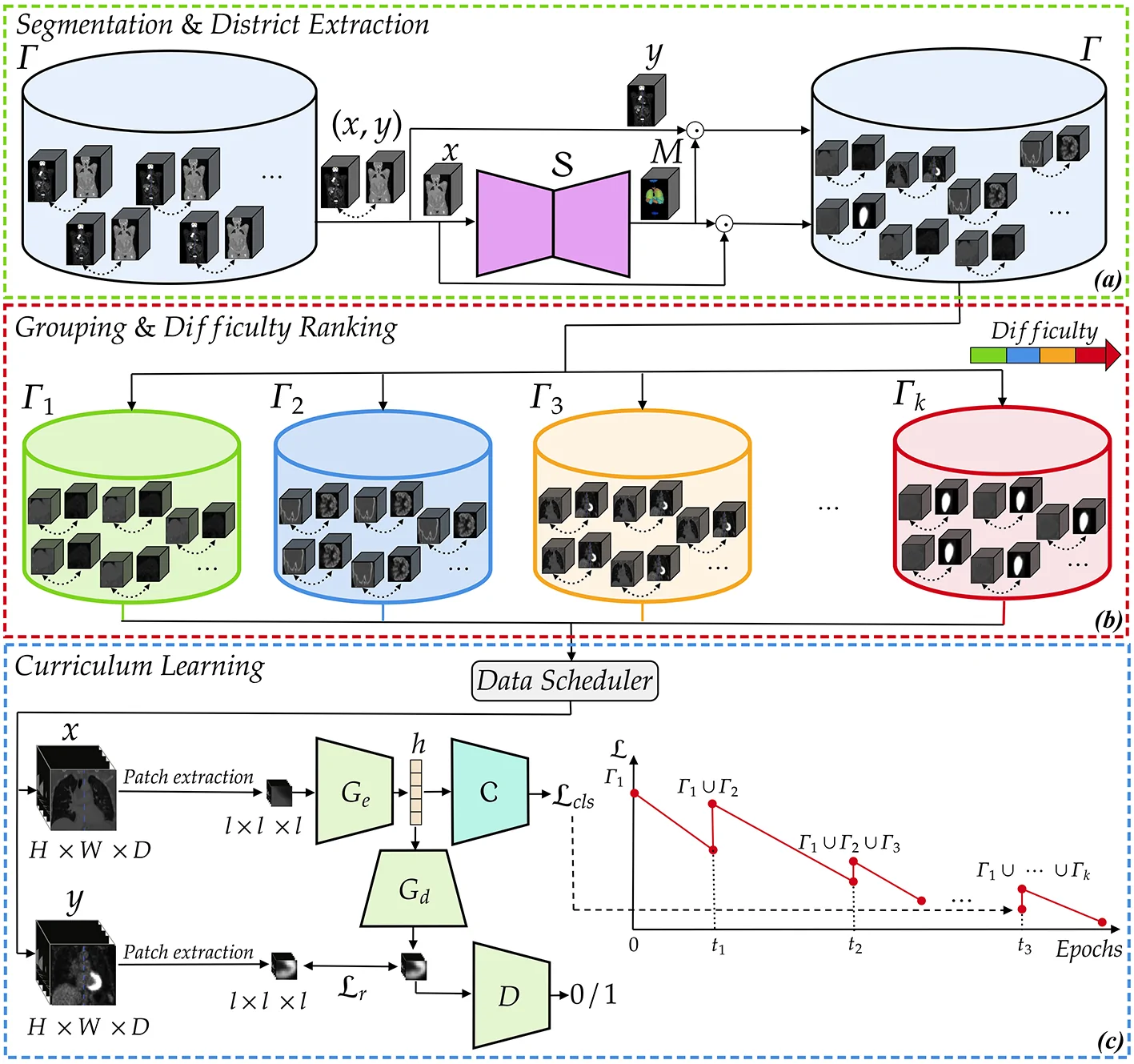
Overall framework of UniPET. **(a)** A segmentation model *S* is applied to each CT scan *x* to produce a multi-label anatomical mask *M*, which is used to extract region-specific subvolumes from paired CT and PET scans. **(b)** Anatomical regions are grouped into subsets Γ_1_, Γ_2_, …, Γ_*k*_ via hierarchical clustering based on intensity range and texture variance, and are ordered by increasing average volume of each region, assuming that larger regions are more challenging to model. **(c)** During training, a data scheduler progressively introduces subsets to the model. A generator *G* = *G*_*d*_°*G*_*e*_ synthesizes PET volumes, while an auxiliary classifier *C* predicts anatomical regions from the latent representation *z*. A discriminator *D* evaluates the realism of the generated output. Curriculum progression is guided by the classifier loss $L_{\text{cls}}$, which decreases as more difficult subsets are introduced.

### 2.1 Anatomical decomposition

The first stage of UniPET, shown in Figure 1a, aims at decomposing whole-body CT scans into anatomically distinct regions. Given a paired dataset Γ={(x,y)|x∈X,y∈Y}, where each pair (*x, y*)∈Γ comprises a CT scan (*x*) and its aligned PET counterpart (*y*), we apply a pretrained segmentation network *S* (Sundar et al., 2022) to each CT volume *x* to obtain the mask *M* = *S*(*x*). It shares the spatial dimensions of *x* and assigns each voxel an integer label *i*∈{1, …, 14}, which corresponds to one of 14 anatomical regions (legs, arms, liver, lungs, brain, stomach, pancreas, spleen, kidneys, trachea, bladder, thyroid, gallbladder, and adrenal glands).

For each anatomical label *i*, we derive a bounding box that tightly encloses the segmentation mask corresponding to region *i*: it defines the minimal 3D ROI that spatially delimits the organ under consideration, thereby reducing irrelevant background voxels and facilitating subsequent region-level analysis. This anatomical decomposition forms the basis for curriculum learning, supporting gradual exposure to spatially and structurally diverse regions. It is worth noting that this segmentation step is only required during training. At inference time, the model operates directly on the full CT volume using a sliding-window approach with patch size *l* = 32 and stride *s* = *l*/2, without relying on segmentation masks. Predictions from overlapping patches are combined by voxel-wise averaging in the shared regions to reconstruct the final whole-body PET volume and reduce discontinuities at patch boundaries. Furthermore, inference requires a single generator and no additional segmentation step, simplifying the whole-body reconstruction pipeline compared with region-specific approaches.

### 2.2 Region grouping and difficulty ranking

In the second stage, we organize the anatomical regions by similarity and by increasing difficulty, as shown in Figure 1b, assuming that structurally similar regions pose comparable learning difficulty and can therefore be introduced at the same stage of training (Bengio et al., 2009; Soviany et al., 2020; Liu et al., 2021). To this aim, for each region *x*_*i*_, we compute two features: the intensity dynamic range and the voxel intensity variance. The intensity dynamic range Δ*d*_*i*_ provides a measure of contrast and it is defined as:

$$\begin{array}{l} \Delta d_{i}=\max\left(x_{i}\right)-\min\left(x_{i}\right), \end{array}$$

The voxel intensity variance $\sigma_{i}^{2}$ is a texture measure given by:

$$\begin{array}{l} \sigma_{i}^{2}=\frac{1}{N_{i}}\overset{N_{i}}{\underset{j=1}{\sum }}{\left(x_{i,j}-\mu_{i}\right)}^{2}, \end{array}$$

where *x*_*i, j*_ denotes the intensity of voxel *j* in region *i*, *N*_*i*_ is the number of voxels in the same region, and μ_*i*_ is the mean intensity:

$$\begin{array}{l} \mu_{i}=\frac{1}{N_{i}}\overset{N_{i}}{\underset{j=1}{\sum }}x_{i,j}. \end{array}$$

Using these two features, hierarchical clustering returns the dendrogram reported in Figure 2a, where we empirically set the cutoff threshold (the red dashed line in the figure) to group the regions by similarity content. It is worth noting that the threshold was selected to retain the most similar region groups while avoiding two opposite configurations: overly large groups would introduce excessive variability early in training, undermining the progressive nature of curriculum learning, whilst overly small groups would fragment the curriculum and reduce its efficiency. The selected threshold yields three region pairs: bladder with trachea, adrenal glands with gallbladder, and pancreas with stomach, while the remaining regions are treated individually.

**Figure 2:**
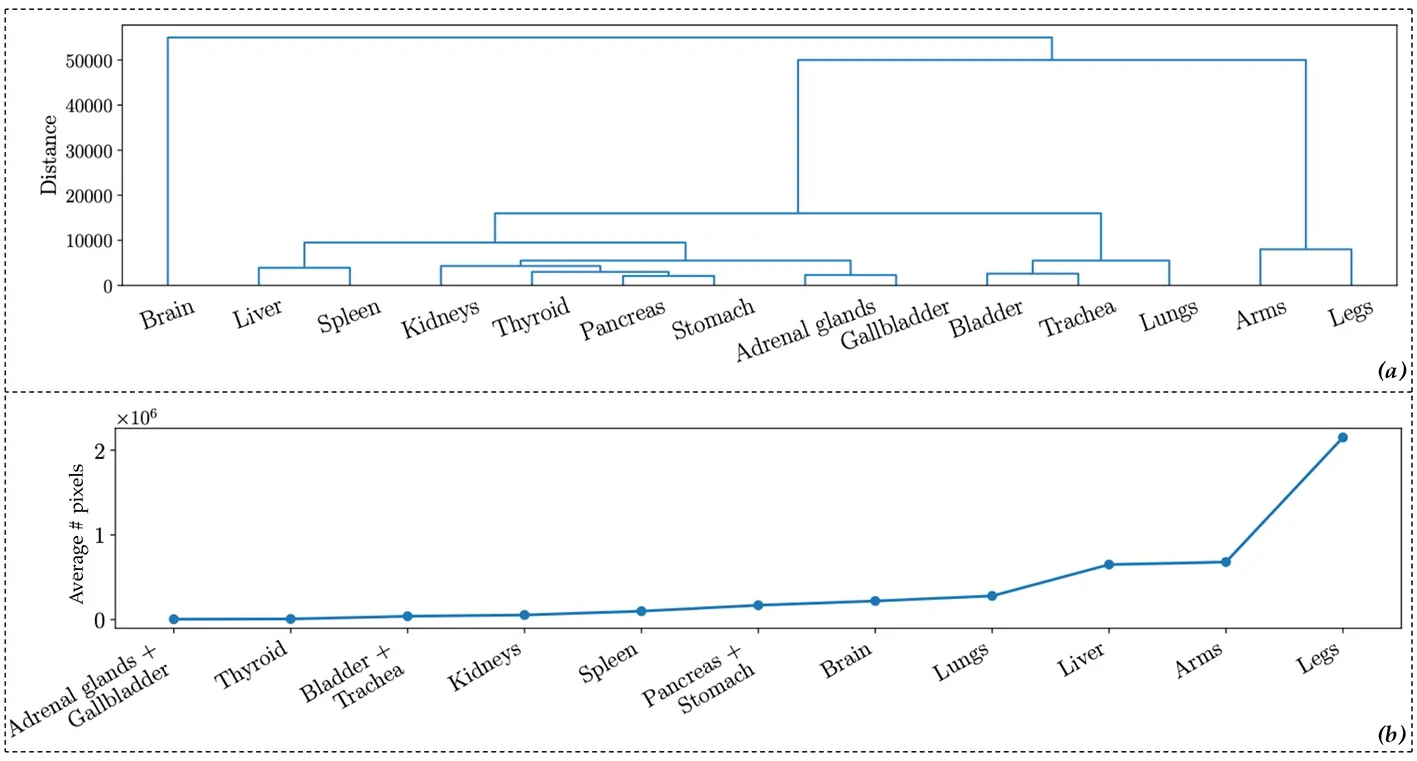
Clustering and difficulty ranking of anatomical regions used for curriculum learning. **(a)** Dendrogram resulting from hierarchical clustering of the 14 anatomical regions based on intensity dynamic range and voxel intensity variance. The red dashed line indicates the empirically selected cut-off threshold, used to define semantically coherent region groups. **(b)** Average number of voxels per anatomical region (or cluster), used to estimate task complexity. Larger regions are assumed to be more difficult to learn due to increased spatial variability and metabolic heterogeneity.

We then organize the grouped regions into subsets Γ_*k*_ by average volume across the training set, which serves as a proxy for modeling difficulty. The use of volume at this stage complements the texture-based clustering described above: while intensity range and variance are used to identify regions with similar image characteristics, volume determines their ordering within the curriculum. Although anatomical volume does not directly measure metabolic complexity, larger regions exhibit greater spatial variability, more heterogeneous tissue composition, and a wider range of anatomical-to-metabolic correspondences, making their associated translation tasks inherently more challenging. The resulting ranking, shown in Figure 2b, defines the scheduling strategy adopted during training.

### 2.3 Curriculum-based CT-to-PET translation

We train a GAN-based CT-to-PET translation model using curriculum learning. A data scheduler progressively introduces anatomical subsets Γ_*k*_ based on their ranked difficulty, allowing the model to first learn mappings in structurally simpler regions and gradually generalize to more complex ones. UniPET builds on a 3D extension of the Pix2Pix architecture (Isola et al., 2017; Pang et al., 2021), in which the generator *G* = *G*_*d*_°*G*_*e*_ consists of an encoder *G*_*e*_, a decoder *G*_*d*_, and an intermediate latent representation *h*. A discriminator *D* assesses the realism of the synthesized PETs, while an auxiliary classifier *C* predicts the anatomical region of each input sample using *h*: its loss function $L_{cls}$ guides the scheduler, with more difficult subsets introduced as classification performance improves. Training is performed on 3D patches of size *l*×*l*×*l*, randomly extracted from both CT and PET volumes to manage computational cost and increase data diversity. Given an input CT patch $x_{i}^{p}\subset x$ and the corresponding PET patch $y_{i}^{p}\subset y_{i}$, the model is trained to minimize the following objective:

$$\begin{array}{l} L=L_{adv}+\lambda_{r}L_{rec}+\lambda_{c}L_{cls}, \end{array}$$

where $L_{adv}$ is the adversarial loss, $L_{rec}$ is the pixel-wise reconstruction loss, and $L_{cls}$ is the classification loss.

The adversarial loss uses a standard GAN formulation, and is defined as:

$$\begin{array}{l} L_{adv}\left(G,D\right)={𝔼}_{x_{i}^{p},y_{i}^{p}}\left[\log D\left(x_{i}^{p},y_{i}^{p}\right)\right]+{𝔼}_{x_{i}^{p},z}\left[\log\left(1-D\left(x_{i}^{p},G\left(x_{i}^{p}\right)\right)\right)\right], \end{array}$$

where *G* and *D* are trained in an alternating min-max fashion, *E* denotes the expectation operator, and *z* is a gaussian noise vector implemented via dropout on the layers of *G*.

The voxel-wise reconstruction loss encourages the generator *G* to synthesize PET patches that are close to the ground truth at the voxel level. It is computed as:

$$\begin{array}{l} L_{rec}={\left\|G\left(x_{i}^{p}\right)-y_{i}^{p}\right\|}_{1}. \end{array}$$

where ‖·‖_1_ denotes the ℓ_1_ distance computed over all the voxels included in the patch *p* of region *i*.

The classifier *C* is trained with a multiclass cross-entropy loss:

$$\begin{array}{l} L_{cls}\left(C\right)=-\overset{14}{\underset{i=1}{\sum }}c_{i}\log{ĉ}_{i} \end{array}$$

where the sum is over the 14 classes and *y*_*i*_ and ŷ_*i*_ are the ground truth region labels and predicted probabilities for region *i*, respectively.

Curriculum progression takes place at discrete stages *t*_*k*_, so that at each stage the training set is incrementally expanded to include all patches from subsets Γ_1_ to Γ_*k*_, i.e., Γ_1_∪⋯∪Γ_*k*_. Transition to the next stage *t*_*k*+1_ is triggered once the classification loss $L_{cls}$ plateaus, indicating convergence on the current subset and, thus, suggesting that the model has adequately captured its underlying characteristics and can be exposed to more complex subsets. To this end, we introduce a dynamic warm-up period *T*_warm-up_, proportional to the size of the newly incorporated regions, during which no transition is allowed. This is followed by a patience interval *T*_patience_, over which the plateau condition is evaluated. They are defined as:

$$\begin{array}{l} T_{\text{warm-up}}=\frac{N_{\text{voxel}}^{k}}{N_{\text{voxel}}^{\text{max}}}\cdot \tau_{1}+\tau_{2}, \end{array}$$

where $N_{\text{voxel}}^{k}$ is the average number of voxels in the Γ_*k*_ newly introduced region, $N_{\text{voxel}}^{\text{max}}$ corresponds to the voxels count of the largest region (legs).

The parameters τ_1_ and τ_2_ are both empirically set to 200 epochs, providing a sufficient adaptation period without excessively slowing curriculum progression; τ_2_ defines the minimum interval, while τ_1_ modulates it according to the relative volume of the newly introduced subset.

## 3 Experimental configuration

### 3.1 Implementation and training details

All networks were initialized using normal weight initialization and optimized with the Adam optimizer (Kingma and Ba, 2014), with β_1_ = 0.5 and β_2_ = 0.999 to ensure stable convergence. A default learning rate of 2·10^−4^ was used with a linear decay schedule. All experiments run on a single NVIDIA A40 GPU. Region-specific and whole-body GANs were trained for a total of 168 h. UniPET was initialized from the whole-body model and trained using the curriculum learning strategy, with the latter stage taking ≈96 h. To ensure comparability, all models used the same architectures, hyperparameters, and training procedures. Training was performed in a patch-based manner with patch size *l* = 32 and a batch size of 512 patches. To improve model robustness and reduce overfitting, we applied data augmentation consisting of random rotations (up to ±10°) along each spatial axis, random flipping across axial, sagittal, and coronal planes, and Gaussian noise injection with standard deviation σ = 0.01. The segmentator S was implemented using MOOSE (Sundar et al., 2022), a pretrained semantic segmentation model, to segment the CT volumes. For statistical analysis, we employed the non-parametric Friedman test to assess overall performance differences among the whole-body GAN, region-specific GAN, and UniPET across anatomical regions. When statistically significant differences were observed, pairwise model comparisons were performed using the Nemenyi *post-hoc* test, which accounts for multiple comparisons, with a significance threshold of *p* < 0.05.

### 3.2 Evaluation metrics

We perform two complementary evaluations to assess the performance of UniPET. The *first* quantifies global image similarity between synthetic and real PET scans through two well-established reference-based metrics that evaluate signal fidelity and structural consistency (Di Feola et al., 2023). The *second* focuses on quantitative metabolic accuracy at the lesion level.

#### 3.2.1 Global image similarity

We assess the overall similarity between real and synthetic PET in terms of intensity preservation and structural correspondence. We use two image quality assessment metrics: Peak Signal-to-Noise Ratio (PSNR) and Structural Similarity Index (SSIM).

PSNR compares the maximum possible intensity in the image ($\text{MA}{\text{X}}_{ŷ}^{2}$) with the error between the synthetic image ŷ and the real reference *y* given by the Mean Squared Error (MSE):

$$\begin{array}{l} \mathrm{PSNR}\left(ŷ,y\right)=10\cdot \log_{10}\left(\frac{{{\text{MAX}}_{ŷ}}^{2}}{\text{MSE}\left(\hat{y},y\right)}\right), \end{array}$$

where the MSE is defined over the 3D volume as:

$$\begin{array}{l} \text{MSE}\left(\hat{y},y\right)=\frac{1}{V_{y}}{\left\|\hat{y}-y\right\|}_{2}^{2} \end{array}$$

with *V*_*y*_ and ${\left\|\cdot \right\|}_{2}^{2}$ denoting the image volume and the ℓ_2_ distance, respectively. PSNR ranges from 0 to +∞ and it is expressed in decibels: higher values indicate better quality, while lower values reflect higher distortion or error in the synthetic image.

SSIM computes the local similarity between two images as a function of luminance, contrast, and structure (Wang et al., 2004):

$$\begin{array}{l} \mathrm{SSIM}\left(ŷ,y\right)=\frac{\left(2\mu_{ŷ}\mu_{y}+c_{1}\right)\left(2\sigma_{ŷy}+c_{2}\right)}{\left(\mu_{ŷ}^{2}+\mu_{y}^{2}+c_{1}\right)\left(\sigma_{\hat{y}}^{2}+\sigma_{y}^{2}+c_{2}\right)} \end{array}$$

where $\mu_{\hat{y}}$ and μ_*y*_ are the mean intensities of pixels in ŷ and *y*, respectively; similarly $\sigma_{\hat{y}}^{2}$ and $\sigma_{y}^{2}$ are the variances, and $\sigma_{\hat{y}y}$ is the covariance. Constants *c*_1_ and *c*_2_ are included to ensure numerical stability. SSIM ranges from 0 to 1, with values closer to 1 indicating higher image quality and structural similarity.

#### 3.2.2 SUV-based quantitative evaluation

While PSNR and SSIM assess visual and structural similarity, they overlook metabolic fidelity, a key characteristic of PET imaging. To this end, we perform a lesion-level comparison of SUV biomarkers computed from real and synthetic PET volumes. We evaluate three well-known complementary measures: *SUV*_*max*_, metabolic tumor volume (*MTV*_α_), and total lesion glycolysis (*TLG*_α_), which collectively quantify metabolic activity in terms of intensity and volumetric extent (Boellaard et al., 2015). Denoting a ROI within the PET as R⊂y, the maximum standardized uptake value *SUV*_*max*_ quantifies peak metabolic activity as

$$\begin{array}{l} {\mathrm{SUV}}_{\text{max}}=\underset{m\in R}{\max}\left(y\left(m\right)\right), \end{array}$$

where *y*(*m*) is the intensity of the voxel m∈R.

The metabolic tumor volume (*MTV*_α_) estimates the extent of metabolically active tissue by counting the voxels exceeding a fixed uptake threshold α = 1.5, which is a well-established value in PET image analysis (Kinahan and Fletcher, 2010; Li et al., 2019):

$$\begin{array}{l} {\mathrm{MTV}}_{\alpha }=|R_{\alpha }|,\;\text{where }R_{\alpha }=\left\{m\in R|y\left(m\right)>\alpha \right\}. \end{array}$$

The total lesion glycolysis (*TLG*_α_) integrates both activity and volume of the active region:

$$\begin{array}{l} {\mathrm{TLG}}_{\alpha }=\left(\frac{1}{\left|R_{\alpha }\right|}\underset{m\in R_{\alpha }}{\sum }y\left(m\right)\right)\cdot {\mathrm{MTV}}_{\alpha }. \end{array}$$

To assess the agreement between SUV-based biomarkers extracted from real and synthetic PET images, we adopt two complementary measures: the intraclass correlation coefficient (ICC) (McGraw and Wong, 1996) and the Spearman's rank-order correlation coefficient (SROCC) (Schober et al., 2018).

The ICC measures absolute agreement between paired measurements by jointly accounting for both correlation and systematic bias, thus assessing whether the biomarkers extracted from synthetic PET are interchangeable with their real counterparts. It ranges from 0 to 1, with higher values denoting stronger agreement.

The SROCC instead measures the strength of the monotonic association between biomarkers extracted from real and synthetic PET, independently of their absolute scale. Unlike the ICC, it is insensitive to linear bias and assesses whether lesion-wise rankings of metabolic activity are preserved. It ranges from −1 to 1, with positive values indicating direct monotonic association, negative values indicating inverse association, and values close to zero indicating little or no monotonic relationship. To quantify the uncertainty associated with these estimates, 95% confidence intervals were reported for both SROCC and ICC.

### 3.3 Datasets and preprocessing

#### 3.3.1 Internal dataset

We conducted all experiments using the FDG-PET-CT-Lesions dataset (Gatidis et al., 2022), a publicly available collection of 1,014 whole-body PET/CT scans from 900 patients. The dataset includes subjects diagnosed with malignant lymphoma, non-small cell lung cancer, and malignant melanoma, as well as healthy controls. Of the total scans, 501 contain at least one annotated malignant lesion, while the remaining 513 serve as negative samples. Within the positive cohort, 168 cases correspond to lung cancer, 145 to lymphoma, and 188 to melanoma. All imaging was acquired using a Siemens Biograph mCT scanner, following a standardized acquisition protocol to ensure consistency across patients. CT volumes were acquired during expiration with arms elevated, using a tube voltage of 120 kV and a tube current of 200 mAs under automated exposure control. Slice thickness ranged from 2.0 to 3.0 mm, with an in-plane resolution between 0.7 and 1.0 mm. Intravenous contrast was administered selectively based on patient weight. PET imaging was performed across multiple bed positions following at least 6 h of fasting, with a mean injected dose of 314.7 MBq. Each patient comprises three series in DICOM format: the CT scan, the corresponding FDG-PET volume, and a binary segmentation mask for tumor lesions. The dataset was split into a training set comprising 864 paired CT-PET scans and an internal test set of 150 paired scans, guaranteeing that patches extracted from a patient study are only in the training or test set.

#### 3.3.2 External dataset

External validation was performed using the ENHANCE.PET 1.6k dataset (Ferrara et al., 2025), a publicly available, multi-center collection of whole-body FDG-PET/CT scans acquired across three medical institutions: the University of Tübingen, University Hospital Leipzig, and Azienda Ospedaliero Universitaria Careggi. The dataset comprises 1,597 PET/CT studies acquired from subjects with lung cancer, lymphoma, and malignant melanoma, as well as individuals without known disease. Imaging data were acquired on different PET/CT systems, including Siemens Biograph mCT, Philips Gemini TF, and GE Discovery MI scanners. Diagnostic CT scans were acquired with tube voltages between 100 and 140 kVp and reconstructed with slice thicknesses ranging from 1 to 5 mm, while PET imaging was performed following intravenous administration of FDG after a minimum of 6 h of fasting, with a mean injected activity of approximately 314 MBq and variable uptake times. All data were anonymized and released in NIfTI format, including paired CT and PET volumes for each subject, together with CT-derived anatomical segmentations of major organs, bones, muscles, cardiac subregions, and body composition compartments. Since data from the University of Tübingen overlap with the data used as the internal dataset by us, they were excluded from the external validation. Thus, the external cohort comprises a total of 579 paired CT-PET scans acquired from University Hospital Leipzig and Azienda Ospedaliera Universitaria Careggi.

#### 3.3.3 Preprocessing

Preprocessing involved conversion to NIfTI format, followed by CT resampling to match the PET resolution. PET voxel values were converted into Standardized Uptake Values (SUV) (Kinahan and Fletcher, 2010), defined as:

$$\text{SUV}=\frac{r\cdot w}{a^{\prime }}$$

where *r* is the radioactivity concentration [kBq/mL] in a region of interest (ROI), *a*′ is the decay-corrected injected activity [kBq], and *w* is the patient's weight [kg], representing the tracer's distribution volume. To account for modality-specific intensity variability, PET volumes were clipped and rescaled to the SUV range [0, 20], capturing the majority of tumor-related values. CT volumes were left unwindowed to preserve full dynamic range across anatomical structures. Finally, both modalities were normalized to the range [0, 1] to improve numerical stability and training convergence.

## 4 Results

### 4.1 UniPET generates accurate and realistic PET-like images

We evaluate the performance of UniPET in synthesizing PET-like images using the peak signal-to-noise ratio (PSNR) to assess signal fidelity and the structural similarity index measure (SSIM) to quantify structural and perceptual similarity (Wang et al., 2004).

Figure 3a shows the distribution of PSNR and SSIM in the 14 anatomical regions using the violin plots, whereas Figure 3b reports the same metrics stratified by diagnostic category. The interested readers will find a table summarizing such performance in Supplementary Tables S1, S2. PSNR attains a maximum of 36.7 dB in the arms, with values across most regions falling between 27 and 35 dB; the lowest performance is observed in the bladder (17.55 dB). We observe a similar trend for the SSIM. With respect to disease stratification, UniPET achieves comparable PSNR performance across lung cancer, lymphoma, melanoma, and negative cases, with values ranging from 29.9 to 30.94 dB, indicating consistent image quality across pathological conditions and limited disease-specific effects. Similar considerations apply to SSIM, suggesting that intensity fidelity and structural similarity are tightly coupled in CT-to-PET translation, and that performance variations are primarily driven by organ-specific physiological complexity rather than metric-specific artifacts. In other words, when the model successfully captures the anatomical-functional correspondence for a given region, both voxel-wise accuracy and structural coherence improve simultaneously, whereas regions characterized by heterogeneous or highly variable tracer uptake exhibit concurrent degradation in both metrics. The evolution of PSNR and SSIM during curriculum-based training is shown in Figure 3c. Although transient fluctuations are observed as additional anatomical regions are progressively introduced, both PSNR and SSIM exhibit a clear upward trend, increasing from initial values of approximately 28.8 dB and 0.64 to about 30.6 dB and 0.73, respectively. Toward the end of training, both metrics stabilize, indicating convergence to a stable and consistent PET synthesis regime.

**Figure 3:**
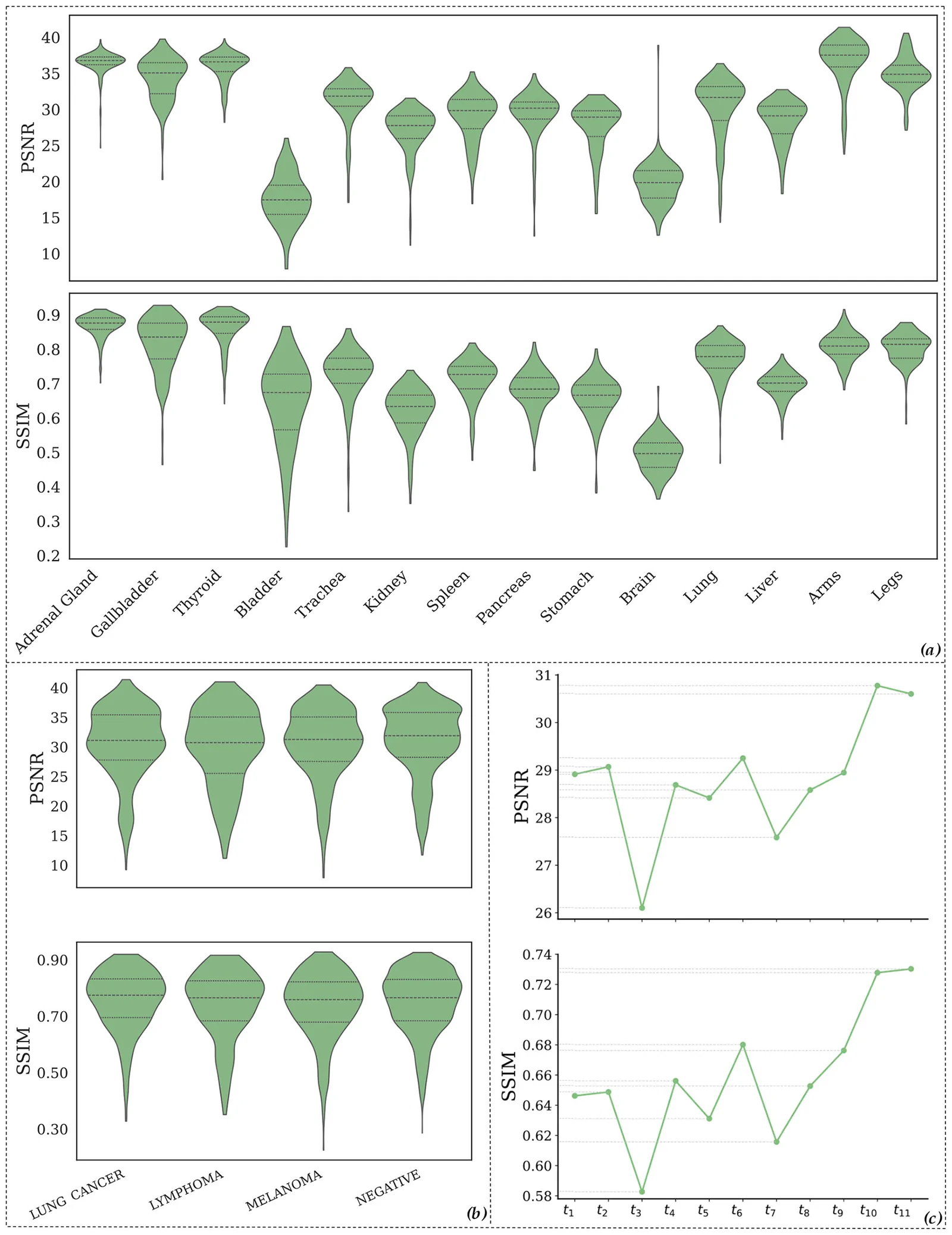
Quantitative and qualitative evaluation of UniPET performance. **(a)** Violin plots showing the distribution of PSNR and SSIM across anatomical regions, comparing real and synthetic PET images. **(b)** Distribution of PSNR and SSIM scores across diagnostic categories. Higher values denote improved signal fidelity and structural similarity, respectively. **(c)** Evolution of PSNR and SSIM across curriculum stages. The plots show a progressive improvement as training advances, indicating that the staged introduction of anatomical regions facilitates learning and enhances overall model performance.

### 4.2 UniPET preserves SUV information

We next evaluated the ability of UniPET to preserve SUV-derived metabolic biomarkers at the lesion level by relying on the manually annotated lesion masks available in the original dataset (Gatidis et al., 2022).

Figure 4a reports Spearman rank order correlation coefficients (SROCC) and intraclass correlation coefficients (ICC) between real and synthetic PET volumes for two representative metabolic biomarkers: *MTV*_1.5_ and *TLG*_1.5_. The former analysis shows that region-specific models achieve the highest rank correlations, reaching 0.67 for *MTV*_1.5_ and 0.61 for *TLG*_1.5_. UniPET follows closely, with correlations of 0.58 and 0.52, respectively, and it outperforms the vanilla whole-body GAN across both biomarkers, indicating a superior preservation of the relative ordering of metabolic activity. Across all methods, SROCC values decrease from *MTV*_1.5_ to *TLG*_1.5_, reflecting the increased sensitivity of TLG to voxel-level intensity variations. ICC provides a complementary assessment of absolute agreement, showing higher values for *MTV*_1.5_ than for *TLG*_1.5_ across all approaches. This behavior is expected, as MTV is volume-dependent, whereas TLG is jointly dependent on both the volume of the lesion and the intensity of uptake. Region-specific models achieve near-unit ICC for *MTV*_1.5_ (0.97), while UniPET attains slightly lower but still high ICC values (0.84), and all methods show reduced ICC for *TLG*_1.5_. In Figure 4b, we present a lesion-level *SUV*_*max*_ threshold consistency analysis stratified by lesion size quartiles, using sensitivity to quantify the preservation of uptake above the reference threshold. Sensitivity increases monotonically with lesion size for all models: in the smallest lesions ( ≤ 25%), region-specific GANs show the highest sensitivity equal to 0.53, while UniPET with 0.47 outperforms the vanilla whole-body GAN. For medium-to-large lesions, i.e, from the second up to the fourth quartile, UniPET closes the gap with region-specific GANs and reaches near-ceiling sensitivity equal to 0.97, often matching or slightly exceeding region-specific models. We show lesion-level ROIs in Figure 4c, highlighting the visual correspondence between real and synthetic PET. As expected, the whole-body GAN largely fails to reproduce intra-lesional metabolic activations, leading to a marked loss of uptake contrast within lesion boundaries. While the region-specific GAN provides the closest match to the ground truth, UniPET closely aligns with it in terms of intra-lesional activity and further exhibits improved preservation of surrounding tissue, resulting in smoother transitions and more coherent uptake distributions beyond the lesion margins.

**Figure 4:**
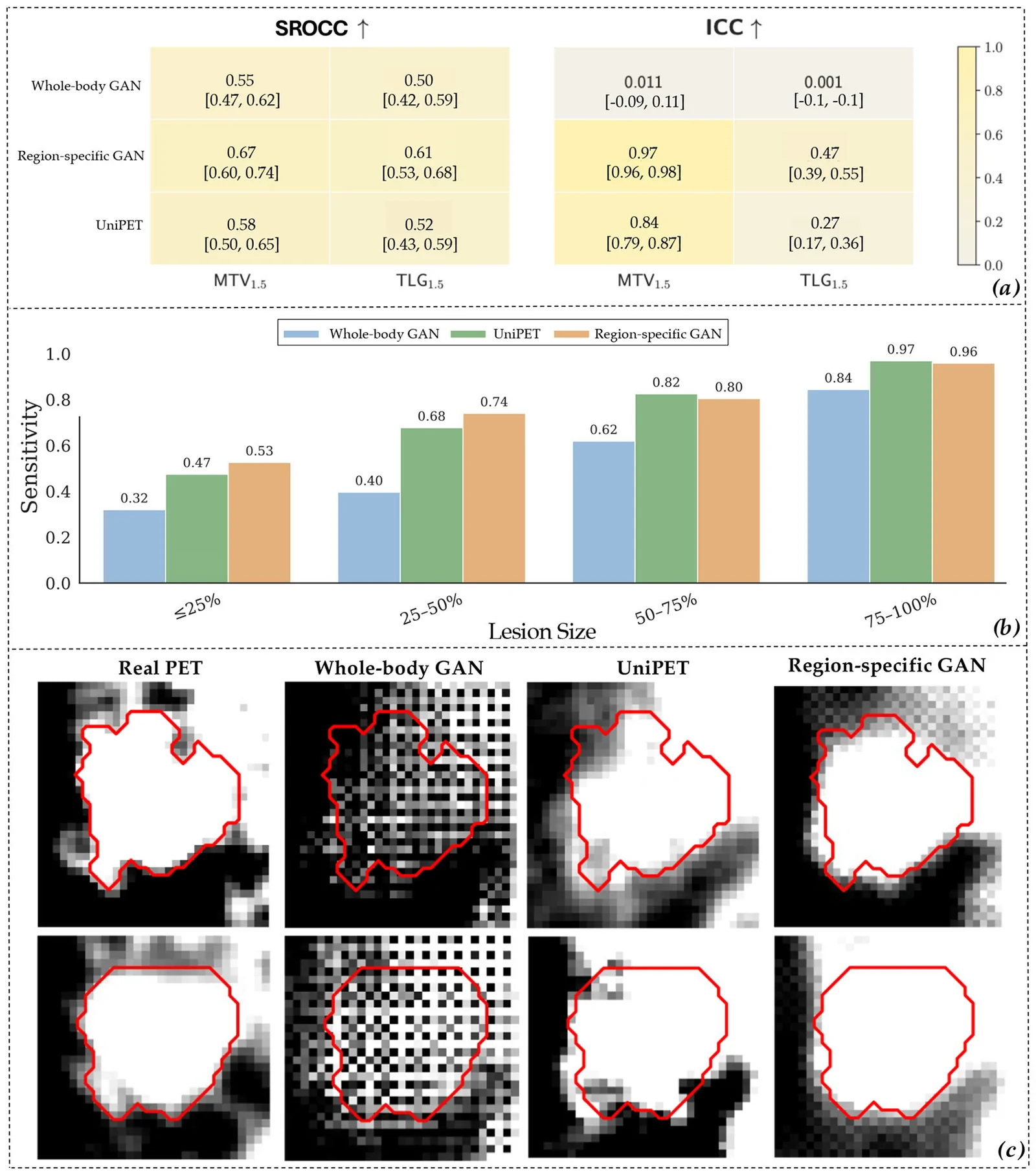
Quantitative evaluation of UniPET's ability to preserve SUV-derived biomarkers. **(a)** Correlation analysis between real and synthetic PET volumes at patient and lesion levels using SROCC and ICC for *MTV*_α_ and *TLG*_α_ across multiple thresholds. Values in brackets indicate the corresponding 95% confidence intervals. **(b)** Lesion-level *SUV*_*max*_ threshold consistency between real and synthetic PET within manually annotated lesion ROIs, stratified by lesion size. **(c)** Representative examples with zoomed ROIs highlighting lesion-level correspondence between real and synthetic PET. *Whole-body* refers to a single GAN trained on whole-body data, whereas *Region-specific* refers to independent GANs trained separately for each anatomical region.

### 4.3 One model or many models?

We compared UniPET with a single vanilla whole-body GAN and a set of region-specific GANs, each independently trained on a single anatomical region to assess the trade-off between model unification and specialization (Figure 5).

**Figure 5:**
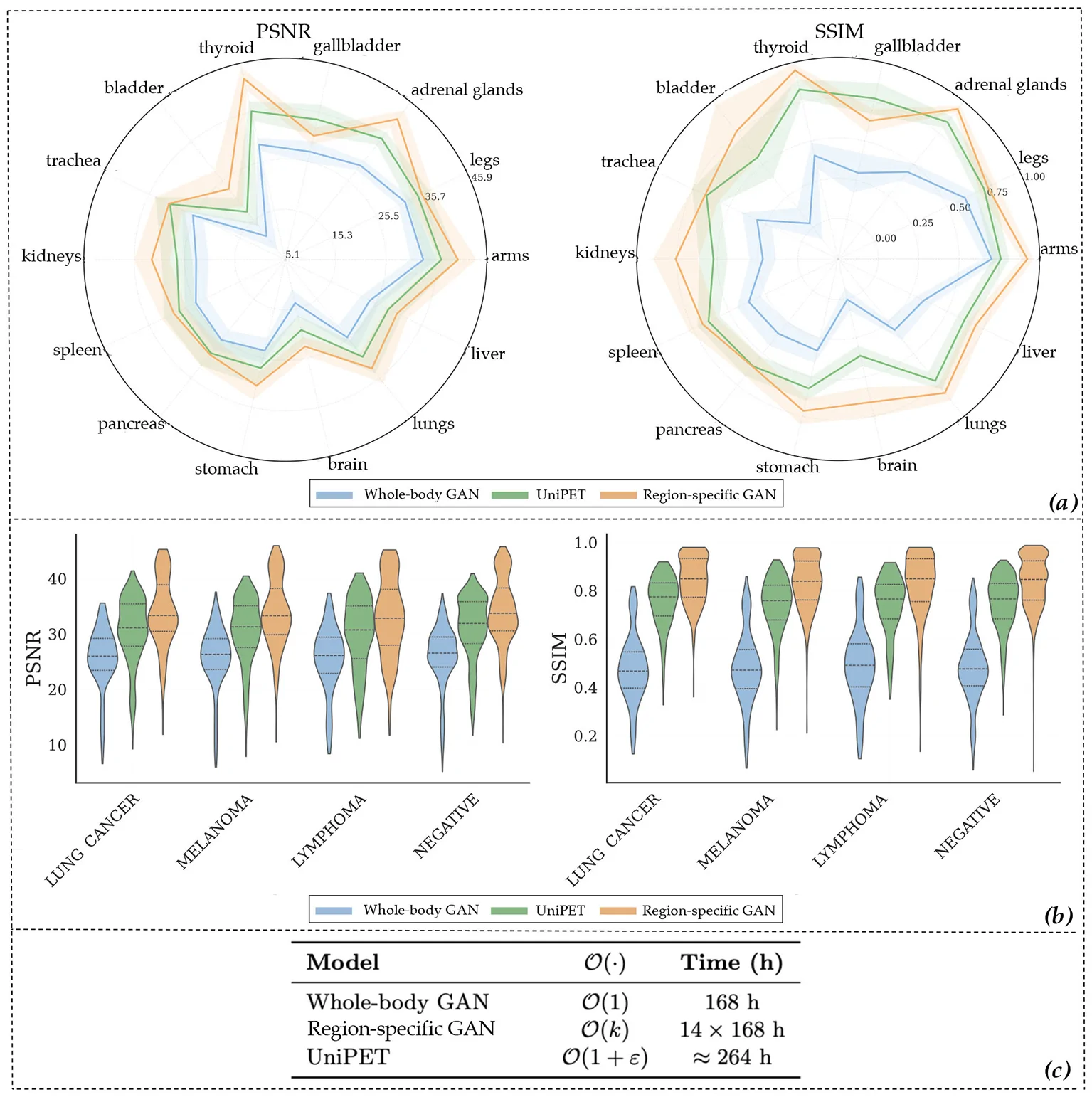
Comparison among whole-body, region-specific, and UniPET approaches. **(a)** shows PSNR and SSIM distributions across anatomical regions; **(b)** reports results across diagnostic categories; **(c)** summarizes computational complexity and training time for each configuration. *Whole-Body* refers to a single GAN trained on whole-body data, whereas *region-specific* refers to independent GANs trained separately for each anatomical region.

Figure 5a presents radar plots comparing PSNR and SSIM across such regions: UniPET follows the performance of region-specific models across them, with no statistically significant differences in PSNR and SSIM (*p*>0.05), whereas the vanilla whole-body shows inferior performance (*p* < 0.05), according to the Nemenyi *post-hoc* test (see Section 3.1). Performance distributions for UniPET remain compact across regions, indicating stable behavior without systematic degradation in comparison to specialized models. The stratification by diagnostic category, shown in Figure 5b, reports PSNR and SSIM in lung cancer, melanoma, lymphoma, and negative cases: we observe that UniPET maintains high image-quality scores across all categories, outperforming the vanilla whole-body model and remaining close to region-specific models, indicating that image quality is preserved independently of disease category.

Turning our attention to the computational cost reported in Figure 5c, training region-specific models scales linearly with the number of organs, whereas UniPET scales as O(1+ϵ), with a total training time of approximately 264 h on a training dataset composed of 864 scans, including ~10^6^ patches.

### 4.4 UniPET generalizes to an independent patient cohort

We evaluated the generalization ability of UniPET using an external publicly available cohort (Ferrara et al., 2025), comprising 579 paired whole-body FDG-PET/CT scans from lung cancer patients collected across two independent hospitals. We evaluate the results using PSNR and SSIM scores computed over the 10 anatomical regions for which segmentation masks are available in the dataset. Moreover, an evaluation based on SUV-derived metabolic biomarkers cannot be performed because lesion annotations are not available.

Figure 6a illustrates the relative percentage variation Δ(%) of PSNR and SSIM between the internal and external test sets for each anatomical region. Positive values indicate a degradation in performance when the model is evaluated on the external cohort relative to the internal test set, whereas negative values denote the opposite. The interested readers will find numerical values of PSNR and SSIM on the external dataset in Supplementary Table S3. We observe that region-specific models decrease both performance scores on the external cohort across multiple organs, whereas the whole-body GAN increases them. In contrast, UniPET has smaller performance variations when applied to the external dataset, so that its performance variations are not statistically significant (*p*>0.05) both in the case of PSNR and SSIM.

**Figure 6:**
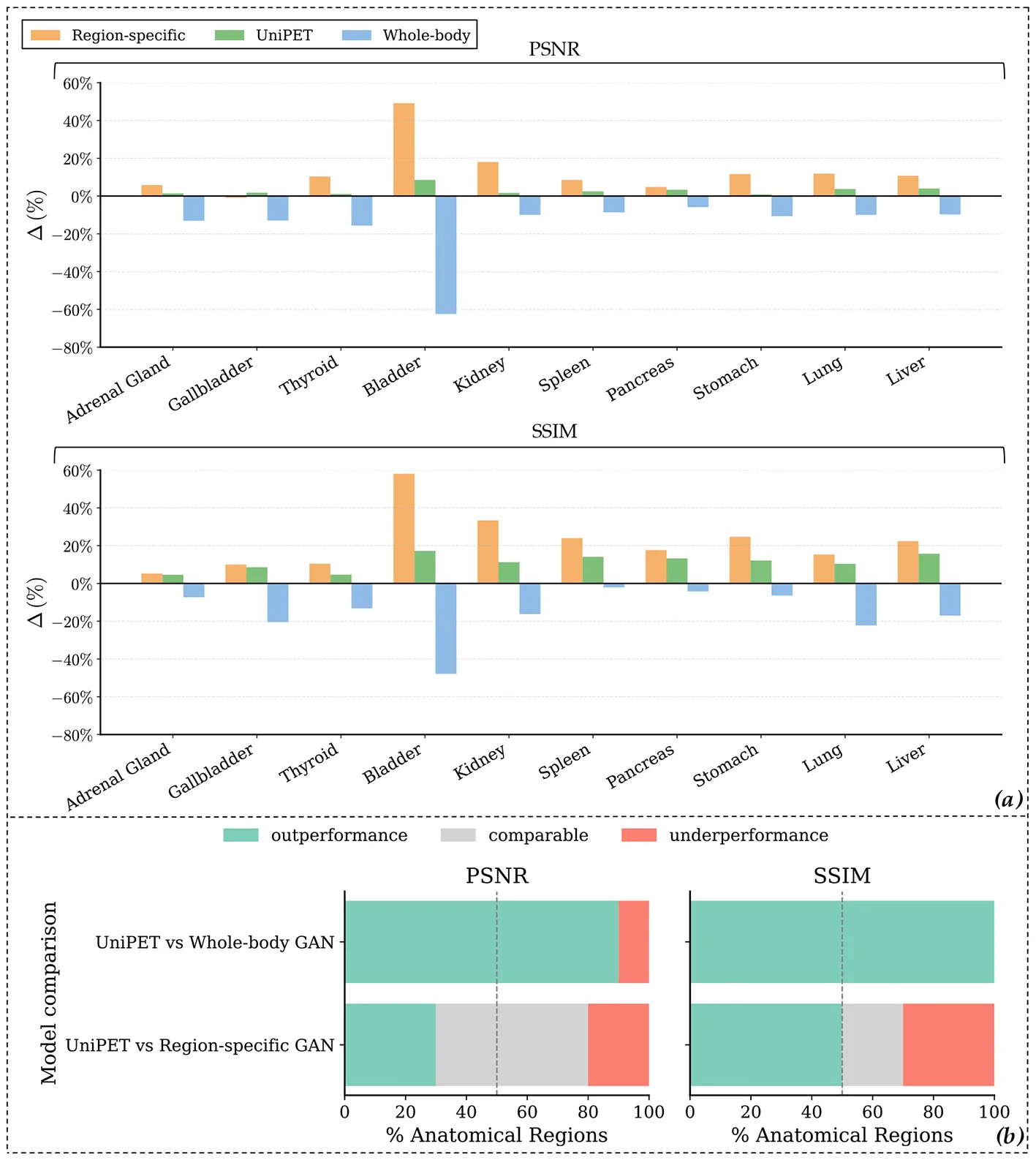
Generalization performance on an independent patient cohort. **(a)** Relative percentage variation Δ(%) of PSNR and SSIM between the internal and external test sets across anatomical regions for region-specific, whole-body, and UniPET models. Positive values indicate performance degradation on the external cohort, while negative values indicate performance improvement. **(b)** Region-level win-tie-loss analysis comparing UniPET against whole-body and region-specific models on the external test set for PSNR and SSIM. A win or loss is assigned when the relative performance difference exceeds ±1%, while differences within ±1% are considered comparable. Bars denote the percentage of anatomical regions falling into each category. *Whole-Body* refers to a single GAN trained on whole-body data, whereas *region-specific* refers to independent GANs trained separately for each anatomical region.

Figure 6b reports a win-tie-loss analysis summarizing the proportion of anatomical regions in which UniPET exhibits superior, comparable, or inferior performance on the external cohort with respect to the whole-body GAN and the region-specific models. For each anatomical region, models are compared pairwise based on PSNR and SSIM; a win is assigned when UniPET achieves a higher average score than its counterpart, a loss when the score is lower, and a tie when the difference falls within a negligible margin (1%). UniPET outperforms the whole-body GAN in the majority of regions (9/10 for PSNR and 10/10 for SSIM) and shows larger performance relative to region-specific models in a subset of regions (9/10 for PSNR and 10/10 for SSIM), indicating stable and competitive region-level behavior on unseen data.

The results reported in Figures 6a, b together suggests that UniPET better generates synthetic PET images across independent cohorts compared with both region-specific and whole-body baselines, reflecting improved robustness to input data variation.

## 5 Discussion

We have introduced UniPET, a unified CT to PET translation framework designed to synthesize whole-body PET images using a single model trained across 14 anatomical regions. Our analysis demonstrates that UniPET captures morpho-metabolic relationships across different regions, while maintaining competitive performance with respect to region-specific models and substantially outperforming a vanilla whole-body GAN approach.

The results in Figure 3a reveal four recurring performance patterns. First, extremities (arms and legs), glands (thyroid and adrenal glands), and gallbladder exhibit high PSNR and SSIM. In these regions, morpho-functional coupling is favorable, as low background uptake and strong air-tissue contrast in CT provide robust constraints for PET synthesis. Second, respiratory organs (trachea and lungs) exhibit lower performance, likely due to respiratory motion, vascular heterogeneity and partial-volume effects in sub-centimetric lesions; however, these factors increase variance without compromising average accuracy. Third, liver, spleen, and kidneys show intermediate yet stable performance, where inter-subject metabolic variability can broaden the distributions. Fourth, bladder and brain emerge as the most challenging regions: bladder due to strong tracer excretion dynamics, whilst brain due to high uptake weakly correlated with CT density. These findings also highlight key differences with respect to prior CT-to-PET translation approaches. Existing methods typically adopt either whole-body models, which tend to smooth out region-specific uptake patterns and struggle to capture local anatomical variability, or region-specific models, which require training and maintaining multiple networks, limiting scalability to whole-body applications. In contrast, UniPET provides a unified framework that achieves performance comparable to specialized models while maintaining a single-model design. This balance between specialization and scalability represents a practical advantage for real-world applications, where maintaining multiple pipelines may be impractical.

The training dynamics under the curriculum learning further clarify how UniPET integrates heterogeneous anatomical information. As shown in Figure 3c, PSNR and SSIM evolve non-monotonically, reflecting the staged inclusion of anatomical regions with distinct statistical and volumetric properties. The curriculum ordering by average region size (Figure 2b) explains these fluctuations: early inclusion of small, low-information regions leads to transient metric drops (Figure 3c), whereas progressively incorporating larger regions (e.g., lungs, liver and extremities) stabilizes optimization by providing richer morpho-structural context. The final convergence toward higher and more stable PSNR/SSIM values indicates that curriculum learning enables UniPET to progressively consolidate a unified CT-to-PET mapping that generalizes well to external data (Figure 6). This is further supported by comparison with a random curriculum ordering (Supplementary Table S4), which results in a performance drop across most anatomical regions. PSNR decreases in nearly all organs, with reductions exceeding 2 dB in several cases (e.g., gallbladder, thyroid, and trachea), and similar trends are observed for SSIM.

Figure 5a shows us that performance distributions remain almost compact across most regions, indicating that UniPET does not incur a systematic penalty compared with region-specific GANs. Indeed, in this figure, we observe that UniPET follows the region-specific models across organs, with PSNR and SSIM differences that are not statistically significant (*p*>0.05), while the whole-body GAN shows significantly inferior performance (*p* < 0.05). Moreover, analyses stratified by diagnostic category (Figures 3b, 5b) confirm this trend: across lung cancer, melanoma, lymphoma and negative cases, UniPET maintains narrow distributions and high PSNR/SSIM, outperforming the whole-body baseline and remaining moderately close to region-specific models. These results suggest that unification does not degrade image quality when changing either organ or pathology, supporting the idea that explicit specialization by region or diagnosis is not strictly required. Nonetheless, in regions with weak morpho-metabolic coupling or high physiological dynamics (e.g., brain and bladder), organ-specific models retain an advantage.

While the discussion reported so far has focused on PSNR and SSIM scores, which measure image fidelity, let us now deepen the quantitative analysis of the metabolic signal. In Figure 4a, the Spearman rank correlation (SRCC) analysis indicates that region-specific models best preserve the relative hierarchy of metabolic activations. We deem this happens because each specialized model is trained on data restricted to a single anatomical organ, thus limiting training data variability compared with UniPET and the whole body GAN. UniPET, although not matching the performance of region-specific models, outperforms the whole-body approach, suggesting that curriculum learning promotes a coherent metabolic ordering across organs rather than relying only on local correlations. The decrease in SRCC from *MTV*_1.5_ to *TLG*_1.5_ across all methods highlights the following challenge: rank preservation becomes increasingly sensitive to small boundary inaccuracies and voxel-level intensity deviations because TLG integrates both volume and intensity. The Intraclass Correlation Coefficient (ICC) provides a complementary perspective by assessing absolute agreement: its values are higher for *MTV*_1.5_ than for *TLG*_1.5_ across all approaches, reflecting the volumetric nature of MTV in contrast to the sensitivity of TLG to intensity scaling. Region-specific models achieve ICC close to 1 for *MTV*_1.5_, denoting precise calibration of uptake values within anatomical regions. For *TLG*_1.5_, ICC decreases across all methods, highlighting the intrinsic challenge of reproducing metabolic measures that jointly integrate uptake intensity and lesion volume. Taken together, the combination of high SRCC and lower ICC demonstrates that UniPET robustly preserves metabolic activations, whereas exact voxel-wise agreement remains a more demanding objective.

Turning to Figure 4b, the *SUV*_*max*_ threshold analysis further characterizes lesion-level uptake preservation. Sensitivity increases with lesion size for all models, reflecting partial-volume effects and noise affecting small lesions. In the smallest volume quartile ( ≤ 25%), region-specific models retain an advantage, as training within a single anatomical domain promotes region-tailored intensity scaling that better preserves high-uptake peaks represented by few voxels. For medium-to-large lesions, UniPET rapidly closes the gap and reaches near-ceiling agreement, often matching or slightly exceeding region-specific models.

From a computational perspective, UniPET achieves a favorable trade-off between performance and scalability. Training complexity scales as O(1 + ϵ), requiring approximately 1.6 × the time of a single whole-body model but nearly an order of magnitude less than training multiple organ-specific models.

At inference time, UniPET requires a single generator and no anatomical segmentation, simplifying whole-body reconstruction compared with region-specific approaches, which rely on multiple generators and an additional segmentation stage. In addition, overlapping predictions are combined through voxel-wise averaging, reducing discontinuities at patch boundaries.

Finally, the evaluation on an external and public cohort (Ferrara et al., 2025), including 579 patients from two independent hospitals, provides direct evidence of the model's ability to generalize beyond the training domain. The win-tie-loss analysis (Figure 6b) shows that UniPET outperforms the whole-body GAN across most anatomical regions, whilst its performance is comparable to region-specific models. Furthermore, the relative performance variation between internal and external test sets reported in Figure 6a of the same figure further clarifies these results. In particular, it is worth noting that region-specific models decrease their performance on the external cohort. We attribute this behavior to their training paradigm: each network is optimized using data from a single anatomical region, thereby being exposed to a narrower distribution of anatomical appearances and metabolic patterns. This reduced variability increases the risk of overfitting to region-specific training characteristics and limits the models' ability to generalize to unseen data, in contrast to UniPET, which benefits from exposure to a broader and more diverse set of anatomical contexts during training. Moreover, the whole-body GAN exhibits an apparent performance improvement on the external test set, a behavior consistent with an underfitting regime in which the learned representation is overly coarse. In this setting, the model captures only generic anatomical-functional correspondences, which generalize uniformly across datasets but remain suboptimal in terms of region-specific fidelity. In contrast, UniPET exhibits a very small performance gap between the internal and external test sets, which is also statistically negligible, indicating improved robustness to distribution shifts. By leveraging curriculum learning, UniPET effectively balances specialization and generalization, avoiding both the overfitting tendencies of region-specific models and the underfitting behavior observed in the vanilla whole-body approach. This training strategy enables the model to learn region-aware representations while preserving exposure to a broad range of anatomical contexts, resulting in more stable and predictable performance across independent cohorts, an essential requirement for reliable clinical deployment. Taken together, these findings emphasize that generalization should not be interpreted solely as maximizing in-domain performance, but rather as the ability to maintain consistent and robust behavior when confronted with previously unseen data distributions. Moreover, a key advantage of UniPET is that it does not require segmentation at inference time. Its unified architecture processes the full volume directly, eliminating the need for the segmentation step used by region-specific models.

From a clinical perspective, synthetic PET should not be regarded as a replacement for acquired PET. Since metabolic information is inferred exclusively from CT, abnormalities with weak or absent anatomical correlates may not be reliably reproduced, while residual uptake differences may affect quantitative biomarker assessment. Therefore, UniPET is not intended for clinical decisions requiring accurate metabolic quantification, such as definitive staging or treatment-response assessment. Rather, synthetic PET may provide complementary functional information for triage-oriented settings or as an additional input for computational tasks, including multimodal scenarios where one or more imaging modalities are unavailable and synthetic modalities can act as surrogate inputs (Aksu et al., 2026). Nonetheless, prospective clinical validation is required before clinical use.

Our work has some limitations that should turn into future research directions. First, UniPET shows reduced accuracy for small lesions and for anatomical regions characterized by weak morpho-metabolic coupling or high physiological dynamics (e.g., brain and bladder), where partial-volume effects and global intensity calibration remain challenging. Second, although curriculum learning stabilizes performance across regions, intensity scaling discrepancies persist, which may affect absolute biomarker quantification and could be addressed through improved calibration. Third, the external validation is limited to a single pathology (lung cancer), reflecting the current scarcity of publicly available paired CT-PET datasets. While this choice allows for reproducible benchmarking, it may limit the generalisability of the findings across other oncological or non-oncological conditions. Future work will therefore aim to extend the evaluation to a broader range of pathologies as more diverse datasets become available. Future work could also focus on size-aware modeling strategies, partial-volume-aware corrections, or *post-hoc* calibration on the synthetic PET scans. Extending UniPET to additional radiotracers, acquisition protocols, and longitudinal settings represents a further promising direction to strengthen its clinical applicability.

## 6 Conclusions

In summary, UniPET advances whole-body CT-to-PET translation by providing a unified approach balancing synthetic image quality accuracy, metabolic fidelity, and computational efficiency. Our findings suggest that UniPET may support the integration of inferred metabolic information into CT-based workflows, particularly in screening and triage settings where PET imaging is impractical or unavailable. This is especially relevant in low-resource environments, where access to PET remains limited; for example, only a few scanners serve large populations across multiple low-income countries (World Health Organisation and Our World in Data, 2022). In such contexts, synthetic PET may provide complementary functional cues to help prioritize cases for further investigation. More broadly, UniPET may also enhance CT-only workflows by providing inferred metabolic context that can be leveraged by downstream analysis pipelines, such as detection, segmentation, or risk stratification models, without requiring dedicated PET acquisition.

## Data Availability

Publicly available datasets were analyzed in this study. This data can be found here: https://www.cancerimagingarchive.net/collection/fdg-pet-ct-lesions/, https://registry.opendata.aws/enhance-pet-1-6k/.
