# Supplementary material for: UniPET: a unified approach for whole-body CT to PET translation
Source: Front Artif Intell. 2026 Oct 1;9:1919564. doi: 10.3389/frai.2026.1919564 (PMC13668879; doi:10.3389/frai.2026.1919564)
Supplement: Supplementary file 1 [file Data_Sheet_1.PDF]

Supplementary Material

1 SUPPLEMENTARY TABLES

Table S1. PSNR and SSIM across anatomical regions for the whole-body, UniPET, and region-specific models on the internal test set. Results are reported as mean ± standard deviation. Regions are ordered according to their volume (Figure 2).

**Region**	**Whole-body GAN**		**UniPET**		**Region-specific GAN**	
	PSNR (↑)	SSIM (↑)	PSNR (↑)	SSIM (↑)	PSNR (↑)	SSIM (↑)
Adrenal gland	29.541 ± 1.152	0.552 ± 0.050	36.474 ± 1.510	0.869 ± 0.032	41.546 ± 2.228	0.951 ± 0.018
Gallbladder	27.573 ± 2.076	0.438 ± 0.097	34.260 ± 2.999	0.816 ± 0.077	35.848 ± 1.681	0.903 ± 0.057
Thyroid	29.048 ± 1.256	0.526 ± 0.065	35.967 ± 1.952	0.862 ± 0.048	42.721 ± 2.237	0.960 ± 0.031
Bladder	11.225 ± 3.350	0.225 ± 0.096	17.555 ± 3.421	0.643 ± 0.123	23.497 ± 4.666	0.810 ± 0.165
Trachea	25.897 ± 2.383	0.446 ± 0.080	31.048 ± 1.396	0.725 ± 0.076	31.420 ± 3.058	0.732 ± 0.068
Kidney	23.185 ± 2.444	0.373 ± 0.063	27.064 ± 3.565	0.618 ± 0.068	30.146 ± 3.084	0.742 ± 0.106
Spleen	25.246 ± 2.420	0.492 ± 0.062	28.982 ± 3.249	0.714 ± 0.058	30.216 ± 2.620	0.747 ± 0.073
Pancreas	25.906 ± 2.348	0.474 ± 0.072	29.317 ± 3.111	0.679 ± 0.059	29.666 ± 2.849	0.682 ± 0.058
Stomach	24.016 ± 2.503	0.466 ± 0.058	27.623 ± 3.404	0.659 ± 0.060	31.278 ± 2.766	0.773 ± 0.063
Brain	14.125 ± 2.721	0.205 ± 0.054	19.709 ± 2.890	0.492 ± 0.050	23.084 ± 1.948	0.728 ± 0.073
Lung	25.257 ± 2.986	0.450 ± 0.058	30.278 ± 4.159	0.772 ± 0.052	33.279 ± 3.704	0.849 ± 0.046
Liver	24.148 ± 1.958	0.472 ± 0.041	28.289 ± 2.897	0.695 ± 0.039	30.256 ± 2.252	0.757 ± 0.040
Arms	33.086 ± 2.556	0.761 ± 0.040	36.733 ± 3.305	0.808 ± 0.039	40.168 ± 3.619	0.939 ± 0.027
Legs	32.066 ± 1.413	0.698 ± 0.055	34.956 ± 2.425	0.803 ± 0.044	35.802 ± 2.129	0.823 ± 0.045

Table S2. PSNR and SSIM across diagnostic conditions for the whole-body, UniPET, and region-specific models. Results are reported as mean ± standard deviation.

2***Diagnosis**	**Whole-Body Model**		**UniPET**		**Region-Specific Model**	
	PSNR (↑)	SSIM (↑)	PSNR (↑)	SSIM (↑)	PSNR (↑)	SSIM (↑)
Lung Cancer	25.581 ± 5.542	0.477 ± 0.141	30.590 ± 5.951	0.755 ± 0.107	33.941 ± 6.310	0.841 ± 0.101
Lymphoma	25.310 ± 6.053	0.489 ± 0.157	29.952 ± 6.575	0.739 ± 0.123	32.898 ± 7.167	0.824 ± 0.131
Melanoma	25.662 ± 5.678	0.479 ± 0.151	30.485 ± 5.956	0.738 ± 0.116	33.724 ± 6.248	0.830 ± 0.110
Negative	25.870 ± 5.550	0.483 ± 0.146	30.940 ± 5.910	0.748 ± 0.110	34.108 ± 6.086	0.834 ± 0.106

Table S3. PSNR and SSIM across anatomical regions for the whole-body, UniPET, and region-specific models on the external test set. Results are reported as mean ± standard deviation. Regions are ordered according to the volume in the internal dataset (Figure 2).

**Region**	**Region-Specific**		**UniPET**		**Whole-Body**	
	PSNR (↑)	SSIM (↑)	PSNR (↑)	SSIM (↑)	PSNR (↑)	SSIM (↑)
Adrenal gland	39.14 ± 3.28	0.90 ± 0.07	35.96 ± 2.32	0.83 ± 0.07	33.41 ± 2.03	0.59 ± 0.04
Gallbladder	31.13 ± 2.33	0.63 ± 0.11	33.66 ± 3.47	0.75 ± 0.12	31.13 ± 3.51	0.53 ± 0.09
Thyroid	38.31 ± 3.24	0.86 ± 0.09	35.57 ± 2.35	0.82 ± 0.08	33.60 ± 1.90	0.60 ± 0.04
Bladder	11.94 ± 2.61	0.34 ± 0.18	16.06 ± 5.50	0.53 ± 0.13	18.24 ± 5.92	0.34 ± 0.13
Kidney	26.40 ± 3.39	0.54 ± 0.18	26.63 ± 3.31	0.55 ± 0.13	25.50 ± 4.17	0.43 ± 0.12
Spleen	27.62 ± 2.96	0.57 ± 0.18	28.26 ± 3.18	0.61 ± 0.15	27.43 ± 3.67	0.50 ± 0.08
Pancreas	28.26 ± 2.60	0.56 ± 0.16	28.35 ± 3.00	0.59 ± 0.13	27.43 ± 3.35	0.49 ± 0.08
Stomach	27.63 ± 3.00	0.58 ± 0.14	27.39 ± 3.04	0.58 ± 0.13	26.57 ± 3.86	0.50 ± 0.08
Lung	29.32 ± 4.74	0.72 ± 0.19	29.16 ± 4.23	0.69 ± 0.15	27.78 ± 4.71	0.55 ± 0.12
Liver	27.00 ± 3.28	0.59 ± 0.19	27.17 ± 2.80	0.59 ± 0.15	26.50 ± 3.64	0.55 ± 0.07

Table S4. PSNR and SSIM across anatomical regions for UniPET trained with a random curriculum ordering on the internal test set. Results are reported as mean ± standard deviation, together with the relative differences (Δ) in PSNR and SSIM compared to UniPET using the curriculum ordering presented in section 2 of this paper.

**Region**	PSNR (↑)	$\Delta(dB)$	SSIM (↑)	Δ
Adrenal gland	36.116 ± 2.154	-0.358	0.795 ± 0.139	-0.074
Gallbladder	31.450 ± 4.254	-2.810	0.656 ± 0.081	-0.160
Thyroid	33.890 ± 2.204	-2.077	0.627 ± 0.050	-0.235
Bladder	16.443 ± 3.334	-1.112	0.588 ± 0.119	-0.055
Trachea	28.360 ± 3.265	-2.688	0.702 ± 0.067	-0.023
Kidney	26.280 ± 2.974	-0.784	0.574 ± 0.068	-0.044
Spleen	28.360 ± 3.265	-0.622	0.702 ± 0.067	-0.012
Pancreas	28.855 ± 2.994	-0.462	0.660 ± 0.056	-0.019
Stomach	27.474 ± 3.415	-0.149	0.654 ± 0.058	-0.005
Brain	14.979 ± 3.196	-4.730	0.246 ± 0.060	-0.246
Lung	30.322 ± 4.173	$+0.044$	0.775 ± 0.054	$+0.003$
Liver	27.917 ± 2.700	-0.372	0.676 ± 0.039	-0.019
Arms	34.793 ± 2.638	-1.940	0.727 ± 0.037	-0.081
Legs	35.404 ± 2.226	-0.448	0.796 ± 0.046	-0.007
